# Effect of exogenous lipids contamination on blood gas analysis

**DOI:** 10.1515/almed-2024-0043

**Published:** 2024-05-07

**Authors:** Giuseppe Lippi, Laura Pighi, Gian Luca Salvagno, Elena Tiziani, Maria Elena Castellini, Roberta Ferraro, Brandon M. Henry

**Affiliations:** Section of Clinical Biochemistry and School of Medicine, University of Verona, Verona, Italy; Clinical Laboratory, Division of Nephrology and Hypertension, Cincinnati Children’s Hospital Medical Center, Cincinnati, OH, USA

**Keywords:** blood gas analysis, errors, syringe, lipids, interference

## Abstract

**Objectives:**

The purpose of this study was to investigate the effects of contamination of venous blood with a lipid-containing solution on parameters measured by a modern blood gas analyzer.

**Methods:**

We collected venous blood from 17 healthcare workers (46 ± 11 years; 53 % women) into three blood gas syringes containing 0 , 5 and 10 % lipid-containing solution. Blood gas analysis was performed within 15 min from sample collection on GEM Premier 5000, while triglycerides and serum indices were assays on Roche COBAS C702.

**Results:**

Triglycerides concentration increased from 1.0 ± 0.3 mmol/L in the uncontaminated blood gas syringe, to 39.4 ± 7.8 and 65.3 ± 14.4 mmol/L (both p<0.001) in syringes with 5 and 10 % final lipid contamination. The lipemic and hemolysis indices increased accordingly. Statistically significant variation was noted for all analytes except hematocrit and COHb in the syringe with 5 % lipids, while only COHb did not vary in the syringe with 10 % lipids. Significant increases were observed from 5 % lipid contamination for pO_2_, SO_2_ and lactate, while the values of pH, pCO_2_, sodium, potassium, chloride, ionized calcium, glucose, hematocrit (10 % contamination), hemoglobin and MetHB decreased. All these changes except lactate and CoHb exceeded their relative performance specifications.

**Conclusions:**

Artifactual hyperlipidemia caused by contamination with exogenous lipids can have a clinically significant impact on blood gas analysis. Manufacturers of blood gas analyzers must be persuaded to develop new instruments equipped with serum indices.

## Introduction

Blood gas analysis is universally regarded as an indispensable diagnostic test for investigating serious metabolic disorders, acid-base imbalance, abnormal oxygenation status and overall impairment of respiratory function [1]. The clinical use of this test has considerably increased during the coronavirus disease 2019 (COVID-19) pandemic due to the frequent acid-base derangements developing in patients with severe SARS-CoV-2 infection and for its strong correlation with the short- and medium-term prognosis [2]. Modern blood gas analyzers provide a wide range of measurable analytes in addition to traditional parameters such as pH, partial oxygen pressure (pO_2_), oxygen saturation (SO_2_) and partial carbon dioxide pressure (pCO_2_), now including electrolytes (potassium, sodium and chloride), total hemoglobin (tHb) and hematocrit (Hct), ionized calcium (iCa^2+^), glucose, lactate, carboxyhemoglobin (COHb) and methemoglobin (MetHb) [3]. The use of blood gas analyzers is particularly suitable for critical healthcare areas such as emergency rooms, intensive care units (both adult and pediatric), dialysis units and operating rooms, where rapid and accurate assessment of basic metabolic and respiratory parameters is essential for appropriate treatment and care [4].

Results of blood gas analysis must be accurate and reliable to be truly useful for the clinical decision making, so that the potential influence of some pre-analytical variables that may affect the measurements must be avoided or minimized [5, 6]. Similarly to conventional laboratory testing, some preanalytical issues in blood gas analysis can also jeopardize the quality of the entire testing procedure. Lipemia is one of the main causes of concern, with significant impact in terms of bias on some clinical chemistry, hemostasis, and hematologic parameters that has been well described in the scientific literature [7].

Although the most recent Clinical and Laboratory Standards Institute (CLSI) Approved Guideline C46A2 for blood gas analysis only states that sample turbidity, such as that caused by hyperlipemia or administration of lipid emulsions, can affect the evaluation of tHb and hemoglobin fractions [8], recent evidence suggests that lipemia may compromise the quality of blood gas analysis by altering the values of some other analytes that can now be measured with modern blood gas analyzers [9]. To this end, this study was planned to investigate the effects of contamination of venous blood with an exogenous lipid-containing solution on a vast array of parameters measured by a modern blood gas analyzer.

## Materials and methods

Venous blood was drawn from 17 members of the staff (mean age; 46±11 years; 53 % women) of the Laboratory Medicine Service at the University Hospital of Verona (Italy). An accessible vein on the upper arms was identified and punctured with a 21G × 3/4″ (0.8 × 20 mm) butterfly device (Safety Blood Collection Set, Gemtier Medical, Shangai, China), initially connected to an evacuated 3.5-mL lithium heparin blood tube (Vacutest Kima, Padova, Italy) to discard residual air present in the tube. Thereafter, venous blood was aspirated within three consecutive 1.0 mL, 0.5 mm × 16 mm heparinized blood gas syringes up to their nominal sampling volume (Arterial Blood Sampling Kit, Smiths Medical ASD IN, Minneapolis, MN, USA). The first syringe was left empty before venous blood sampling, while the second and third were pre-filled with 0.05 and 0.10 mL of a lipid-containing solution (Lipidem 200 mg/mL; B. Braun Medical, Sheffield, UK) (Table 1), thus yielding to 5 and 10 % final sample contamination. The specific composition of this lipid emulsion is as follows: 10 % medium-chain triglycerides, 8 % soya-bean oil (long-chain triglycerides, mainly omega-6) and 2 % omega-3 fatty acids (long-chain triglycerides), thus averaging a 20 % triglycerides contents. The range is usually comprised between 0.7 and 1.5 lipids per kg of body weight per day. Immediately after collection the three syringes were capped and mixed by gently rotating between the palms of the hands for approximately 20 s, for enabling an accurate mix of venous blood, lithium-heparin and lipid solution present in the second and third syringes. No dead space was present in any of the collected syringes, thus excluding the presence of ambient air.

**Figure 1:**
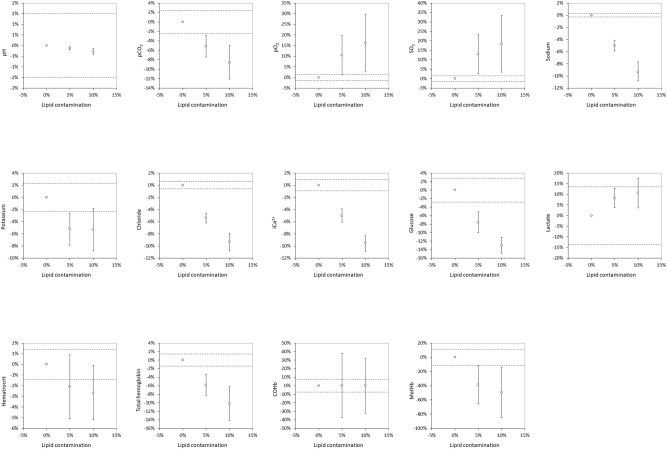
Impact of venous blood contamination with a lipid-containing solution (5 and 10 % contamination) on blood gas analysis. Results are presented as mean bias (and 95 % confidence of interval; 95 %CI) compared to the reference uncontaminated blood gas syringe. The dotted lines represent the (±) performance specifications for each analyte tested. pCO_2_, partial pressure of carbon dioxide; pO_2_, partial oxygen pressure; sO_2_, oxygen saturation; iCa^2+^, ionized calcium; COHb, carboxyhemoglobin; MetHb, methemoglobin.

Blood gas analyses were performed between 12 and 15 min after collection, using the same blood gas analyzer and an identical lot of test cassettes (GEM Premier 5000, Instrumentation Laboratory, Monza, Italy). The concentration of triglycerides and the serum indices were measured on a Roche COBAS C702 (Roche Diagnostics, Basel, Switzerland) after separation of lithium-heparin plasma by centrifugation. The results of measurements were reported as mean and standard deviation (SD). The bias between the reference syringe (i.e. the syringe without lipid contamination) and those containing 0.05- and 0.10-mL lipid solution was expressed as mean variation in test results that exceeded the performance specifications suggested by Kuster et al. [10] (Table 2). Significance of variations of analytes concentration due to the presence of lipid-containing solution were assessed with paired Student’s T-test, while the percent bias from the reference uncontaminated blood gas syringe was calculated with Bland and Altman plot analysis. Statistical significance was set at p<0.05. The statistical analysis was performed using Analyse-it (Analyse-it Software Ltd, Leeds, UK).

All subjects recruited for this study provided a written informed consent for being included. The study was performed in accordance with the Declaration of Helsinki and relevant local legislations and was approved by the Ethics Committee of the University Hospital of Verona (approval number: 970CESC; July 20, 2016).

## Results

The results of our study are summarized in Table 2. The mean concentration of triglycerides increased from 1.0 ± 0.3 mmol/L in lithium-heparin plasma of the uncontaminated blood gas syringe, to 39.4 ± 7.8 mmol/L (p<0.001) in the syringe with 5 % lipid contamination, up to 65.3 ± 14.4 mmol/L (p<0.001) in the syringe with 10 % lipid contamination. Accordingly, the lipemic index (L-Index) also increased from 10.6 ± 3.9 in the uncontaminated blood gas syringe, to 679.2 ± 164.0 (p<0.001) and 1209.8 ± 346.1 (p<0.001) in those with 5 and 10 % lipids. Interestingly, the icteric index (I-index) did not vary significantly in the three blood gas syringes, while the hemolysis index (H-index) increased significantly from 6.1 ± 3.5 in the uncontaminated blood gas syringe, to 23.2 ± 6.0 (p<0.001) and 43.9 ± 20.7 (p<0.001) in the syringes with 5 and 10 % lipid contamination.

**Table 1:** Protocol of the study for evaluating the impact of venous blood contamination with a lipid-containing solution on blood gas analysis.

Syringe	Blood filling volume	Lipid-containing solution	Fractions of blood and lipids
1st syringe	1.0 mL	0 mL	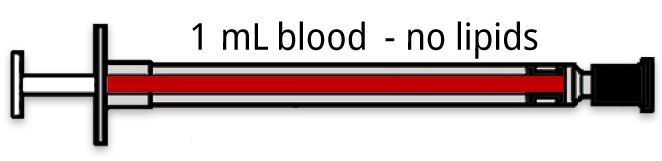
2nd syringe	0.95 mL	0.05 mL	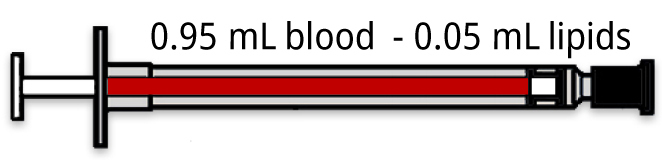
3rd syringe	0.90 mL	0.10 mL	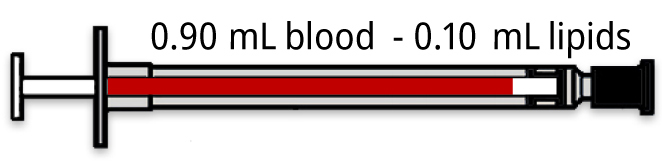

**Table 2:** Impact of venous blood contamination with a lipid-containing solution (5 and 10 %) on blood gas analysis. Test results are presented as mean and standard deviation (SD).

Analyte	Performance specification	Intra-assay imprecision^a^	No contamination	5 % lipid contamination		10 % lipid contamination	
			Value	Value	p-Value^b^	Value	p-Value^b^
pH	±1.5 %	±0.1 %	7.35 ± 0.02	7.34 ± 0.02	0.003	7.33 ± 0.02	0.001
pCO_2_, mmHg	±2.4 %	±1.4 %	49.0 ± 4.9	46.5 ± 4.4	<0.001	44.9 ± 3.4	<0.001
pO_2_, mmHg	±1.5 %	±1.7 %	31.1 ± 10.3	34.4 ± 11.7	0.029	36.6 ± 12.2	0.020
SO_2_, %	±1.5 %	±1.2 %	51.7 ± 17.7	57.8 ± 16.6	0.018	61.3 ± 16.9	0.019
Sodium, mmol/L	±0.3 %	±0.3 %	136.9 ± 0.9	130.2 ± 2.5	<0.001	124.9 ± 3.9	<0.001
Potassium, mmol/L	±2.3 %	±1.0 %	4.33 ± 0.32	4.11 ± 0.27	0.001	4.10 ± 0.22	0.005
Chloride, mmol/L	±0.6 %	±0.4 %	103.2 ± 1.0	97.8 ± 1.7	<0.001	94.0 ± 2.9	<0.001
iCa^2+^, mmol/L	±0.9 %	±1.4 %	1.26 ± 0.06	1.20 ± 0.05	<0.001	1.15 ± 0.06	<0.001
Glucose, mmol/L	±2.8 %	±2.5 %	5.5 ± 0.6	5.1 ± 0.6	<0.001	4.8 ± 0.5	<0.001
Lactate, mmol/L	±13.6 %	±7.5 %	1.11 ± 0.25	1.20 ± 0.27	0.001	1.22 ± 0.24	0.005
Hct	±1.4 %	±3.6 %	45.1 ± 4.0	44.2 ± 4.5	0.162	43.9 ± 4.1	0.041
tHb, g/L	±1.4 %	±2.0 %	148 ± 15	140 ± 14	<0.001	134 ± 15	<0.001
COHb, %	±7.5 %	±43.7 %	1.57 ± 0.96	1.61 ± 0.99	0.937	1.60 ± 1.16	0.996
MetHb, %	±11.3 %	±9.3 %	0.89 ± 0.22	0.65 ± 0.29	0.008	0.61 ± 0.37	0.001
Triglycerides, mmol/L	–	–	1.0 ± 0.3	39.4 ± 7.8	<0.001	65.3 ± 14.4	<0.001
H-index	–	–	6.1 ± 3.5	23.2 ± 6.0	<0.001	43.9 ± 20.7	<0.001
L-index	–	–	10.6 ± 3.9	679.2 ± 164.0	<0.001	1209.8 ± 346.1	<0.001
I-index	–	–	19.8 ± 7.7	20.4 ± 8.2	0.491	20.7 ± 8.4	0.364

^a^Intra-assay imprecision of the analyzer calculated on routine venous samples. ^b^Compared to the reference blood gas syringe without lipid contamination. 95%CI, 95 % confidence interval; pCO_2_, partial pressure of carbon dioxide; pO_2_, partial oxygen pressure; sO_2_, oxygen saturation; iCa^2+^, ionized calcium; Hct, hematocrit; tHb, total hemoglobin; COHb, carboxyhemoglobin; MetHb.

As concerns the parameters of the blood gas analysis, a statistically significant variation was noted for all analytes except Hct and COHb in the syringe with 5 % lipid-containing solution, while only COHb did not vary significantly in the syringe with 10 % lipids. As shown in Table 2 and Figure 1, significant increases were observed from 5 % lipid contamination in the concentration of pO_2_, SO_2_ and lactate, while the values of pH, pCO_2_, sodium, potassium, chloride, iCa^2+^, glucose, Hct (only in the syringe containing 10 % lipids), tHb and MetHB decreased. When these changes were compared to the performance specifications [10], all changes exceeded their relative thresholds except for lactate and COHb (Figure 1).

## Discussion

Blood gas analysis makes it possible to obtain important clinical information on metabolism, acid-base balance and oxygenation status, which are crucial for the diagnosis and treatment of a variety of pathological conditions, especially those related to respiratory and metabolic disorders [11]. A high level of quality throughout the blood gas analysis process – from sample collection to data reporting – is therefore crucial, as unreliable test results can have a detrimental effect on patient care [12]. To this end, healthcare providers should strive for high quality, starting with the fact that blood samples are collected correctly and their content is representative of the patient’s actual *in vivo* conditions.

While this concept has been straightforwardly acknowledged for many laboratory-based tests (e.g., clinical chemistry, immunochemistry, hemostasis, hematology), the potential interference from contamination of venous blood with lipids in a blood gas syringe is much less appreciated and investigated in the current scientific literature, although it appears to be relatively common. A recent study by Liu et al. showed that nearly 6 % of all blood gas samples obtained at their institution had clinically significant levels of the L-index [13]. In a previous study in which all blood gas samples were systematically screened for the presence of hemolysis, lipemia, and icterus, Salvagno et al. found a much higher frequency of lipemic specimens, which was almost twice as high (i.e., 11 %) [14]. The problem is hence tangible and likely underestimated, as blood gas samples are processed without centrifugation, thus making it impossible to optically identify the presence of high concentrations of common interfering substances such as cell-free hemoglobin, bilirubin and lipids, which can then generate a substantial bias in many parameters measured by modern blood gas analyzers. The procedures commonly used in clinical laboratories to correct or mitigate the interference caused by lipemia (e.g. precipitation with cleaning solutions, dilution, ultracentrifugation) cannot be used here, as most blood gas analyses are performed outside the laboratory and the operators are unaware that a high lipid concentration may be present when samples are processed using whole blood. Moreover, in case of fluid contamination, these approaches would not be able to resolve the dilutional effect, so that and it would be more appropriate to indicate that samples with suspected contamination by intravenous fluids should be rejected, both in the central laboratory and in POCT.

In a previous study, Jara-Aguirre et al. [9] pooled residual lithium heparin arterial blood gas samples within 60 min of collection with total parenteral nutrition 20 % lipid emulsion (blood contamination between 0 and 15 %), and then measured a number of parameters using a Radiometer ABL90 FLEX blood gas analyzer (Radiometer Medical ApS, Denmark). It was found that the acceptable error criteria were exceeded when lipid emulsion contamination was ≥1 % for pH (reduction), pO_2_ (increase) and pCO_2_, (reduction), when contamination was ≥5 % for sodium (decrease) and potassium (increase), and ≥10 % for iCa^2+^ (increase) and tHb (decrease), respectively. Unlike this previous study, in which the samples were pooled and processed more than 1 h after collection, we used individual samples and all blood gas tests were completed within 15 min of collection. This allowed us to adhere to current CLSI guidelines [8], which state that blood gas analysis should preferably be performed no more than 30 min after the blood gas syringe is drawn. We also employed a newer lipid emulsion compared to the older one used by Jara-Aguirre et al. (Intralipid, Fresenius Kabi, Uppsala, distributed by Baxter Healthcare Corporation, Deerfield, US) [9], which is no longer available in some countries. These important differences between the two study designs may then explain some discrepancies [9]. In fact, although we also observed a reduction of pCO_2_, sodium and tHb, along with an increase of pO_2_, in our study the concentration of iCa^2+^ and potassium was progressively reduced as the lipid contamination increased. Irrespective of these differences, which may be also attributable to the composition of the lipid-containing solution, the most important evidence emerged from these two studies is that the concentration of blood gasses in the syringe may be significantly and even clinically biased when blood is contaminated by a lipid-containing solution, thus potentially resulting in a misleading diagnostic conclusion and inappropriate treatment.

As specifically concerns our results, the variation of some analytes was predictable, since the observed bias was mainly due to the dilution effect of the lipid-containing solution. This is particularly true for the reduced concentration of sodium, chloride, iCa^2+^, tHb, glucose, Hct and tHb. The change in potassium, on the other hand, deserves special focus. Interestingly, although its concentration was found to decrease in both lipid-contaminated syringes (−5.2 and −5.3 %, respectively), the reduction of its concentration in the syringe with 10 % lipids was less than expected due to the dilution effect alone. Thus, additional factors may have contributed to the bias in the measured concentration of this analyte, and *in vitro* hemolysis is the most likely explanation. This is consistent with the fact that we observed a lipid-dependent increase in H-index in centrifuged plasma, reflecting the presence of erythrocyte (osmotic) damage and breakdown likely due to the extremely elevated lipid concentration in the specimens [15]. This is not surprising, as the fact that hyperlipidemia (especially hypertriglyceridemia) can induce *in vitro* hemolysis was demonstrated almost 20 years ago by Dimeski et al. [16]. Regarding the variations in blood gasses, we can exclude possible artifacts due to sample manipulation because, at variance with the study by Jara-Aguirre and colleagues [9], we did not spike residual blood gas specimens with lipids, but the blood gas syringe was prefilled with the lipid-containing solution before blood was drawn. This minimized the risk of introducing air bubbles. Therefore, the more plausible explanation is that the sustained hyperosmolarity caused by the very high concentrations of triglycerides in the samples may have led to erythrocyte hypoxia and a shift towards anaerobic metabolism, which is then associated with lower oxygen consumption and increased lactate generation [17], as observed in our study.

Although we acknowledge that using venous blood to avoid major injury and discomfort caused by an arterial puncture in our cohort of ostensibly healthy volunteers may be viewed as a study limitation, along with the fact that lipid solution *in vitro* does not truthfully reproduce hyperlipidemia *in vivo*, our findings indicate that artifactual hyperlipidemia (mostly hypertriglyceridemia) caused by contamination from exogenous lipids may have a clinically significant impact on blood gas analysis. Because identifying lipemia within a blood gas syringe is challenging, we strongly encourage the manufacturers of blood gas analyzers to engage in developing new instrumentation equipped with the serum indices. Although that the close relationship between dilution and variation of most analytes observed in our study should enable to rule out the possibility of analytical interference, we could not exclude that some degrees of interference could have been involved in the elevation of hemolytic index, blood lactate or partial gas pressure.
